# PPAR*γ* and Its Role in Cardiovascular Diseases

**DOI:** 10.1155/2017/6404638

**Published:** 2017-01-24

**Authors:** Mini Chandra, Sumitra Miriyala, Manikandan Panchatcharam

**Affiliations:** Department of Cellular Biology and Anatomy, Louisiana State University Health Sciences Center, Shreveport, USA

## Abstract

Peroxisome proliferator-activated receptor Gamma (PPAR*γ*), a ligand-activated transcription factor, has a role in various cellular functions as well as glucose homeostasis, lipid metabolism, and prevention of oxidative stress. The activators of PPAR*γ* are already widely used in the treatment of diabetes mellitus. The cardioprotective effect of PPAR*γ* activation has been studied extensively over the years making them potential therapeutic targets in diseases associated with cardiovascular disorders. However, they are also associated with adverse cardiovascular events such as congestive heart failure and myocardial infarction. This review aims to discuss the role of PPAR*γ* in the various cardiovascular diseases and summarize the current knowledge on PPAR*γ* agonists from multiple clinical trials. Finally, we also review the new PPAR*γ* agonists under development as potential therapeutics with reduced or no adverse effects.

## 1. Introduction

Peroxisome proliferation-activated receptor gamma (PPAR*γ*) is a ligand-activated transcription factor from the nuclear receptor family of peroxisome proliferator-activated receptors (PPARs). They contain a ligand binding domain which is hydrophobic and a type II zinc finger DNA-binding domain [1]. PPARs bind as heterodimers with the retinoid X receptor (RXR*α*) which is 9-cis retinoic acid receptor. PPAR RXR heterodimers transactivate genes by binding specific sequences in the promotor regions of these genes. When a ligand activates PPAR*γ* it results in subsequent activation of target genes as well as inhibition of the inflammatory response of transcription factors. In the absence of ligands, this PPAR RXR heterodimer binds to co-repressors and in turn suppresses the target genes [2, 3]. There are four isoforms of PPAR*γ* detected in humans, PPAR*γ*1, PPAR*γ*2, PPAR*γ*3 and PPAR*γ*4 [4]. Out of these isoforms PPAR*γ* 1, 3 and 4 encode the same protein whereas PPAR*γ*2 is expressed in adipose tissue only [5]. The location of human PPAR*γ* gene has been identified as chromosome 3p25 [6]. In mice, the location is on chromosome 6 [7].

PPAR*γ* is activated by both natural and synthetic ligands such as derivatives of prostaglandins like 15-deoxy-Delta12, 14-prostaglandin J2 [8], derivatives of fatty acid oxidation hydroxy octadecadienoic acid (HODE) which are components of oxidized low density lipoproteins (LDL) [9], lysophosphatidic acid (LPA) [10], Thiazolidinediones (TZD) like pioglitazone and rosiglitazone [8] and natural dietary substances found in the food [11].

PPAR*γ* is highly expressed in adipocytes [12], vascular smooth muscle cells (VSMCs) [13], macrophages [14], cardiomyocytes [15] and endothelial cells. PPAR*γ* activation serves a role in glucose homeostasis and adipogenesis in subcutaneous fat [16], regulating the metabolism of lipid in adipocytes, keeping oxidative stress in check as well as inhibiting apoptosis and maintaining endothelial function, cell proliferation and cell differentiation [17]. It also has a role against inflammation [18]. PPAR*γ* activation results in reduced expression of factors such as TNF-alpha, IL-1 and resistin which are insulin resistance-inducing adipokines. In macrophages, PPAR*γ* suppresses the inducible nitric oxide synthase (iNOS) upregulation and reactive oxygen species (ROS) production. These roles serve to benefit against many diseases which are risk factors for cardiovascular disorders such as atherosclerosis, diabetes, hypertension, obesity and dyslipidemia.

## 2. PPAR*γ* and Insulin Resistance

Insulin resistance such as seen in Type 2 diabetes, impaired glucose tolerance, and metabolic syndrome is a well-established risk factor for cardiovascular disease. PPAR*γ* controls the genes encoding peptides or proteins involved in insulin resistance. PPAR*γ* activators are commonly used in the treatment of type 2 diabetes showing certain abnormalities which are associated with the risk of cardiovascular disease such as increased glucose, insulin and triglyceride levels along with reduced levels of high-density lipoprotein cholesterol (HDL-C) and adiponectin levels [19], a hormone produced in white adipose tissue which has antioxidative, anti-inflammatory and vasodilator effects [20] and has been linked to cardiovascular diseases, insulin resistance states and obesity [21]. Other abnormalities include increased circulating levels of non-esterified fatty acids (NEFA) which are implicated in oxidative stress and induction of inflammatory response in the endothelium. They are associated with endothelial dysfunction and hypertension. High NEFA levels may also predispose cardiomyocytes to a ventricular arrhythmia [22].

PPAR*γ* redistributes triacylglycerol from the subcutaneous fat and visceral fat [23] where the activators of PPAR*γ* move the fat from the visceral adipose tissue, liver and muscles towards subcutaneous tissue by increasing insulin sensitivity in the peripheral and hepatic tissue thereby lowering the concentrations of plasma fatty acid [24]. There also occurs an improvement in the glycemic control. There also occurs improvement in the above-mentioned risk factors of diabetes-related risk of cardiovascular diseases such as lowering of triglycerides and plasma NEFA through its effect on the macrophages along with the increased HDL-C and adiponectin [25].

**Figure 1:**
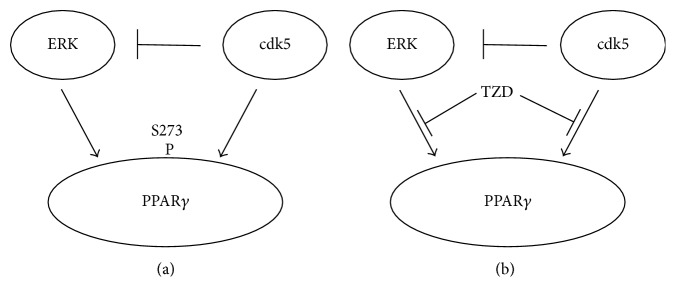
(a) Phosphorylation of PPAR*γ* by ERK and CDK5 which also suppresses ERK Kinase. (b) TZD blocking the access of ERK and CDK5.

The most common activators of PPAR*γ* are Thiazolidinedione (TZD) such as pioglitazone and rosiglitazone which are synthetic agonist ligands of PPAR*γ*. One of the mechanisms of action of TZD is the prevention of the phosphorylation of PPAR*γ*. High-fat diet activates the protein kinase cyclin-dependent kinase 5 (CDK5) activity which leads to the subsequent phosphorylation of PPAR*γ* decreasing its insulin sensitizing effect. For the development of diabetes, PPAR*γ* gets phosphorylated by CDK5 at serine 273 leading to alterations in many genes in the adipose tissue resulting in increased insulin resistance [26]. Recently, the role of CDK5 and extracellular signal-regulated kinases (ERK) was demonstrated in the phosphorylation of PPAR*γ* by Banks et al. They created CDK5 knockout mice in the adipose tissue and demonstrated that in the absence of CDK5, there still occurs an increase in the phosphorylation of PPAR*γ* at serine 273 due to direct effect of ERK. In the presence of CDK5, ERK is suppressed due to its action on ERK kinase (MEK) [27] (Figure 1(a)). TZDs block the phosphorylation of PPAR*γ* by both CDK5 and ERK (Figure 1(b)).

Their data sheds new light to the regulation of PPAR*γ* and another alternative to the treatment of Type 2 Diabetes.

TZDs are already used in the treatment of type 2 diabetes [28]. They are used as monotherapy or as add-on therapy and result in improved insulin sensitivity demonstrating reduced insulin concentrations along with a decrease in the hemoglobin A1c (HbA1c) and fasting blood glucose [25, 29]. TZD also has been shown to increase serum levels of HDL-cholesterol and decrease triglycerides and LDL-cholesterol levels [30]. The decrease in NEFA is observed in both fasting and postprandial levels with decrease becoming apparent as early as 4 weeks of starting treatment [31], with the two TZDs rosiglitazone and pioglitazone showing similar reductions but greater when compared to treatments with metformin, sulfonylureas or statins [32]. TZD treatment doubles the concentration of circulating adiponectin produced by adipose tissue in insulin resistant states [21]. In the diabetic heart, Rosiglitazone demonstrates a protective role, by decreasing cardiac fibrosis and protection against apoptosis as well as improvement in the left ventricular diastolic dysfunction [33, 34]. Similarly, Pioglitazone showed an improvement in the worsening of ischemic preconditioning in the diabetic myocardium [35]. Though the role of TZDs as an insulin-sensitizing treatment conferring benefit to the cardiovascular system would, in theory, be of advantage in future treatments of cardiovascular events associated with high insulin resistance states, clinical trials have not been able to support it. In the BARI-2D (Bypass Angioplasty Revascularization Investigation 2 Diabetes) trial which hypothesized that insulin-sensitizing treatment using TZD would result in greater cardiovascular benefit, lower mean HbA1c and fasting insulin levels was shown although it did not show decreased the occurrence of myocardial infarction (MI) or death upon follow-up 5 years later [36]. It is, however, notable that in the trial the use of multiple glycemic control agents makes it impossible to comment on the efficacy of the TZDs. As mentioned previously, the demonstration by Banks et al. of the involvement of ERK pathway in the phosphorylation of PPAR*γ* in mice model [27] may offer an alternative to increasing the sensitivity of insulin as well lowering the occurrence of cardiovascular events by adding a kinase inhibitor thus increasing the effectiveness of TZDs.

## 3. Atherosclerosis, Vascular Disease and PPAR*γ*

The proliferation of vascular smooth muscle cells and damage to endothelial cells resulting in the expression of adhesion molecules and ultimately leukocyte adhesion are important events in the development of atherosclerosis. Insulin resistance is implicated in the development of atherosclerosis [37]. There are many studies which demonstrate the beneficial role of PPAR*γ* in limiting the progression of atherosclerosis as well as the acceleration of atherosclerosis with the knockout of PPAR*γ* in macrophages [25]. PPAR*γ* ligands are expressed in the atherosclerotic plaques [38] and have an effect on both these cells. Ligands of PPAR*γ* decrease cytokines such as nitric oxide synthase, IL-6, and tumor necrosis factor *α* [14] thereby reducing the inflammatory response associated with atherosclerosis. The secretion of metalloproteinases (MMPs) especially MMP-9, by macrophages is responsible for the rupture of atherosclerotic plaques by degrading the extracellular matrix. PPAR*γ* in both vascular smooth muscle cells and macrophages reduces the expression of MMP thereby hindering the migration of vascular smooth muscle cells thus preventing the plaques from becoming vulnerable to rupture [13, 14]. In 2000, Li et al. demonstrated the inhibition of atherosclerosis progression in LDL receptor-deficient mice using rosiglitazone. The reduction in the atherosclerotic lesion was seen in male mice but did not show similar results in female mice [39]. This highlights the importance of conducting more gender specific studies for actions of PPAR*γ*. To determine whether the improvement seen by Li et al. was due to effect of TZD on the artery itself or on the metabolic system, Collins et al. used LDL receptor-deficient male mice and fed them two different diets, one group was fed high-fat diet and the other group was on high fructose diet, along with 3 months treatment with troglitazone, a PPAR*γ* agonist. The results showed a decrease of the lesion in both groups but only the high-fat diet group of mice showed an increase in insulin sensitivity. Thus the conclusion can be made that the role of TZD in decreasing insulin resistance and decreasing atherosclerotic plaques are independent of each other [40]. In 2009, Nakaya et al. reported prevention of atherosclerotic progression with pioglitazone treatment though the existing lesion was not reversed nor was any improvement seen in advanced atherosclerotic lesions in mice models of LDLR receptor deficiency (LDLR^−/−^) [41]. In 2011, reduction in lesion inflammation was demonstrated in rabbits given pioglitazone treatment for 3 months [42].

Insulin resistance is associated with the development of atherosclerosis. The risk of occurrence of an atherosclerotic event is related to the severity of hyperglycemia as observed by the HbA1c levels [43]. Though in clinical trials such as the PROACTIVE (Prospective Pioglitazone Clinical Trial in Macrovascular Events) a PPAR*γ* activator such as pioglitazone did not show a correlation between its effect on HbA1c and risk of development of cardiovascular disease [44].

Effects of PPAR*γ* agonist on carotid atherosclerosis has been varied. A double blind study done on patients with normal glucose tolerance and stable coronary artery disease showed TZD, pioglitazone stimulates the production of endothelial progenitor cells in vascular injury promoting endothelial repair [45]. The STARR (Study of Atherosclerosis with Ramipril and Rosiglitazone) study compared the carotid artery medial thickness progression in rosiglitazone group compared with placebo. After a study period of 3 years there was a trend towards less carotid artery intimal thickness progression in the rosiglitazone group, however, it was not statistically significant [46]. Another trial comparing carotid artery intimal thickness between two study groups on pioglitazone verses glimepiride (Chicago trial) showed stable carotid artery intimal thickness in pioglitazone group, however, progression of intimal thickness was seen in the glimepiride group which was statistically significant [47]. One long-term study of pioglitazone compared with glimepiride has shown the reversal of carotid atheroma volume in the pioglitazone group. In contrast, the carotid artery atheroma showed progression in the glimepiride group (PERISCOPE trial; Pioglitazone Effect on Regression of Intravascular Sonographic Coronary Obstruction Prospective Evaluation) [48]. Though not conclusive, these studies taken together do point towards the beneficial effect of TZDs. Statins, which are used in the treatment of atherosclerosis result in an increase in PPAR*γ* activity by activating extracellular signal-regulated kinase (ERK) 1/2 and p38 mitogen-activated protein kinase (MAPK) pathways [49]. Lobeglitazone is a new PPAR*γ* agonist shown to have anti-atherosclerotic properties. It can be used in the treatment of patients with a cardiovascular disease associated with diabetes. A significant decrease in the atherosclerotic lesion was observed in apolipoprotein E gene knockout mice (Apo^−/−^) on high cholesterol and high-fat diet with the use of Lobeglitazone, as well as reduced formation of neointima after balloon injury to the carotid artery [50].

Endothelial PPAR*γ* regulates the gene expressions of NADPH oxidase, superoxide dismutase and catalase thus increasing vasodilation [51]. The treatment with TZD resultant activation of PPAR*γ* in adipocytes and inflammatory cells in the adipose tissue inhibits release of inflammatory mediators along with the reduction in local inflammation [52]. ROS can lead to alteration of vascular function [53] thus playing a role in the development of cardiovascular disorders. Oxidative stress reduces the expression of PPAR*γ* in the vascular endothelial cells [54]. In turn, PPAR*γ* has a protective effect on the cardiomyocytes by upregulation of the antiapoptotic Bcl-2 protein against oxidative stress [55].

It is worth noting that the direct effect of PPAR*γ* on the heart leads to heart failure. This was demonstrated in transgenic mice models of PPAR*γ* created by Son et al., which expressed PPAR*γ*1 in the heart. An increase in triglyceride uptake and increased fatty acid oxidation was observed and the mice developed dilated cardiomyopathy with the production of damaged mitochondria [56] when treated with PPAR*γ* agonist TZD rosiglitazone.

## 4. Ischemia-Reperfusion Injury and PPAR*γ*

Previous studies using rat models of ischemia-reperfusion injury have demonstrated a reduction in myocardial damage with the use of TZDs [57]. The TZDs rosiglitazone, pioglitazone, and ciglitazone, all resulted in a decrease of myocardial infarct size.

**Figure 2:**
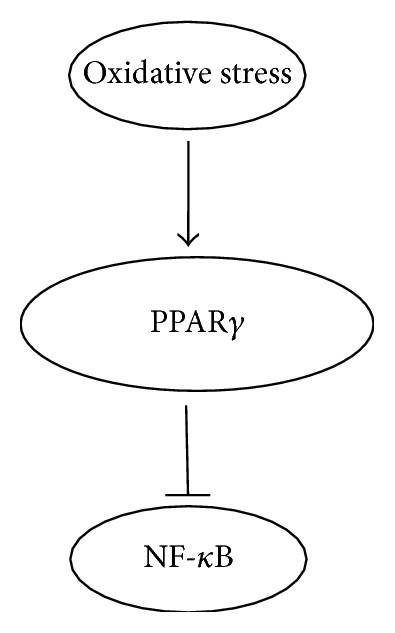
PPAR*γ* suppresses the activation of NF-*κ*B decreasing its inflammatory effects.

Rosiglitazone has a cardio protective effect in both non-diabetic and diabetic rats limiting the damage to the heart following ischemia/reperfusion injury via inhibition of Jun NH (2)-terminal kinase phosphorylation [58]. Another mechanism by which Rosiglitazone provides cardioprotection is by selective overexpression of angiotensin type 2 along with the inhibition of p42/44 MAPK pathway. This effect was demonstrated to be separate from the insulin-sensitizing effect of Rosiglitazone [59]. Yet another mechanism for reduction of heart injury due to ischemia was observed in hypercholesterolaemic rats. Rosiglitazone reduced the increased activity of myeloperoxidase induced by hypercholesterolemia thus resulting in a decrease in infarct size [60]. The TZD ciglitazone results in decreased myocardial damage, infiltration of neutrophils and cytokine production by increasing DNA binding of PPAR*γ* and decreasing the activation of nuclear factor *κ*B (Figure 2) [61]. Pioglitazone also has protective effect against MI, exhibiting reduced infarct size in rabbit model via activation of PPAR*γ*, PI-3 kinases, eNOS and Akt pathways [62].

## 5. Limitation of PPAR*γ* as a Therapeutic Measure in Cardiovascular Disease

Though treatment with activators of PPAR*γ* seems to have a favorable effect on the risk factors for cardiovascular disease, it also has adverse effects on the cardiovascular system which mitigates its beneficial effects thus limiting their widespread use in patients with cardiovascular risk.

In humans, the treatment with PPAR*γ* agonist TZD leads to increase the risk of developing edema and congestive heart failure which is thought to be due to the retention of salt and water (Rubenstrunk, Hanf et al. 2007). The mechanism by which TZD causes fluid retention may be due to the increased transcription of SGK1 (Serum/Glucocorticoid-Regulated Kinase-1) which activates the renal epithelial sodium channels [63]. Currently, the increase in vascular permeability due to increased levels of vascular endothelial growth factor in these patients is known [64]. In a study by Tang et al., no association was observed between severity of heart failure and risk of fluid retention [65], though much research still needs to be conducted before making use of TZDs in clinical settings. PPAR*γ* are also responsible for weight gain due to increase in the adipose tissue mass [66], may increase low-density lipoproteins cholesterol concentration [67], promote the onset of ventricular fibrillation in severe ischemia [68], and modify cardiac ion channels promoting arrhythmia [69]. Even though it has been shown to decrease the progression of atherosclerosis [70], pioglitazone has also been shown to develop plaque necrosis [71] in advanced atherosclerosis in a study done on LDL receptor-deficient mice. It has been reported that treatment with rosiglitazone is linked with an increase in MI in humans [72, 73]. Though in the RECORD (Rosiglitazone evaluated for cardiovascular outcomes in oral agent combination therapy for type 2 diabetes) trial, rosiglitazone was shown to be linked with risk of heart failure and not MI [74]. Unlike rosiglitazone, pioglitazone does not induce cardiac hypertrophy as seen in mice [75]. Pioglitazone has a more positive effect than rosiglitazone on lipid profile with improvement in LDL and triglycerides in patients with diabetes and dyslipidemia [67]. It has also shown a positive effect on the endothelial progenitor cells by increasing their number in patients with coronary artery disease which help in improving vascular function [45]. Due to the possibility of developing cardiac dysfunction with the use of TZD in patients with congestive heart failure, their use in these patients is avoided.

Patients with congestive heart failure are prone to develop heart failure following PPAR*γ* therapy as a result of increased plasma volume. It is notable however that in both animal and clinical studies the resultant heart failure has not been linked to the effect of TZD on left ventricular systolic function [76]. More recently, TZD has been associated with bone loss [77], with the use rosiglitazone shown to be responsible for an increase in fractures in diabetic patients [78].

## 6. Clinical Trials to Determine the Efficacy of PPAR*γ* Activators

### 6.1. IRIS Trial

The IRIS (insulin resistance intervention after stroke) trial is a randomized trial to determine the efficacy of pioglitazone in patients with no history of heart failure, who are insulin resistant and non-diabetic, having a history of recent transient ischemic attack or ischemic stroke. Insulin resistance criteria was an HOMA-IR (Homeostatic Model Assessment-Insulin Resistance) index higher than 3. The hypothesis of this trial was that Pioglitazone will decrease the rate of myocardial infarction and stroke in the selected group. The results of this trial are demonstrated lowered rates of myocardial infarction, stroke, and death in patients receiving pioglitazone. These subjects showed improvement in blood pressure, improved levels of triglycerides, HDL cholesterol and insulin sensitivity. Moreover, the rate of heart failure in the pioglitazone group was not higher than the placebo group. This trial provides information which may be of great importance due to the fact that this trial is in patients who are not diabetic thereby reducing the chances of confounding results by multiple diabetic therapies. This study also determines whether the use of pioglitazone in patients on statins is beneficial.

## 7. PROACTIVE

PROACTIVE (Prospective Pioglitazone Clinical Trial in Macrovascular Events) trial compared pioglitazone with a placebo in the type 2 diabetic patients with HbA1c greater than 6.5% who were also suffering from the atherosclerotic disease in a double-blind study, with a mean follow-up of 34.5 months. It sought to determine the effect of pioglitazone in reducing the incidence of macrovascular complications. Pioglitazone did not have a significant effect on the primary and main secondary endpoints in the patients treated with insulin though there was a lowering in the mean insulin dose in patients on pioglitazone as compared to those on placebo with discontinuation of insulin in 9% pioglitazone patients as compared to 2% placebo patients [79]. Furthermore, in a subgroup analysis done previously, pioglitazone significantly reduced the occurrence of MI by 28% in patients with prior history of MI [80]. Although pioglitazone did significantly reduce the risk of myocardial infarction, it also increased the risk of edema in the subjects. These studies demonstrate a positive efficacy of pioglitazone in cardiovascular disease but a loner observation would be more conclusive. Also, due to the use of other treatments for diabetes in an imbalanced manner in terms of frequency and dosage may also have affected the outcome of study if they also affect the cardiovascular system. Another point of note in these trials is the use of statin in only half of the patients studied. The risk of death was reduced only by 5% in patients treated with a statin and given pioglitazone but was 25% in patients not on statin [44].

## 8. RECORD Trial

RECORD trial tested the use of rosiglitazone as an add-on therapy in patients with type 2 diabetes not controlled by sulfonylurea or metformin alone with mean HbA1c of 7.9%. The group treated with rosiglitazone exhibited higher levels of LDL-C, HDL-C and weight and lower levels. After a mean follow-up of 5.5 years, the group with rosiglitazone had a higher frequency of heart failure. The higher use of statins and diuretics in patients on rosiglitazone as well as the lower event rate and subsequent low statistical power due to lack of follow-up are limitations of this trial [81]. The RECORD results do not show a significant difference between metformin/sulfonylurea group and rosiglitazone group in terms of myocardial infarction, stroke or cardiovascular death. Although the risk of heart failure is relatively small, it is nevertheless an important concern. This reinforces the importance of not using TZDs in patients with heart failure.

## 9. DREAM Trial

The DREAM (Diabetes Reduction Assessment with Ramipril and rosiglitazone Medication) trial was done to determine the effect of 8 mg per day rosiglitazone and/or Ramipril on patients with impaired glucose tolerance or impaired fasting glucose but with no history of cardiovascular disease. Follow-up after 3 years showed a significant reduction in the development of new onset diabetes in patients on rosiglitazone although it was also associated with increased development of heart failure as compared to patients on Ramipril [82]. This study demonstrates a positive role of rosiglitazone in reducing or even eliminating the risk of developing diabetes in obese subjects. However, the short follow-up period of this trial, as well as exclusion of cardiovascular disease history in subjects, limit the conclusions that can be drawn about rosiglitazone and its cardiovascular effects. The heart failure observed in the patients receiving rosiglitazone may be due to the effect of the drug on the kidney resulting in fluid overload a subsequent heart failure in some individuals.

## 10. ACT NOW Trial

The ACT NOW (Actos Now for the prevention of Diabetes) trial sought to determine the effect of pioglitazone in the prevention of new-onset diabetes in patients with impaired glucose tolerance. Patients either received placebo or 45 mg of pioglitazone. After a 2.2-year mean follow-up, a significant reduction in the fasting glucose levels was observed in the pioglitazone group, as well as reduced levels of HbA1c and increase in the levels of HDL-C. 5% of people progressed to diabetes in the pioglitazone group as compared to 16.7% seen in the placebo group. However, the pioglitazone group did have the adverse effect of increased incidence of edema and weight gain [83]. Therefore, in patients with impaired glucose tolerance, pioglitazone reduced the risk of diabetes, improved HDL cholesterol levels and liver enzymes but was associated with edema and weight gain.

## 11. ADOPT Trial

A Diabetes Outcome Progression Trial (ADOPT) used patients with newly diagnosed type 2 diabetes to determine the glycemic durability of three drugs to be used as first-line treatment namely, rosiglitazone, metformin, and glyburide. Treatment was for a median of 4 years with primary outcome set as the failure of monotherapy with fasting blood glucose level more than 10 mmol/L after 6 weeks of treatment. Follow-up at 5 years revealed a lower number of failure in rosiglitazone-treated patients (15%) with low HbA1c and high insulin sensitivity though again was associated with weight gain and edema [84]. This study was significant because it demonstrates that rosiglitazone maintained targeted sugar level for a longer period when compared to metformin and glyburide.

## 12. Metanalysis of Clinical Trials

The meta-analysis of controlled trials of pioglitazone shows a reduction in the risk of MI, stroke or death in patients with type 2 diabetes mellitus [85, 86]. On the other hand, a meta-analysis of trials with rosiglitazone, it has been linked to an increased risk of MI though the associated mortality is still low [87]. Due to this reason, rosiglitazone use is limited in the United States whereas it is not used in Europe [88].

## 13. Comparative Analysis

No randomized trial has been conducted to compare the two TZDs pioglitazone and rosiglitazone although it has been compared in cohort studies, treatment with rosiglitazone is associated with higher rates of cardiovascular events [89–91]. Upon meta-analysis of observational studies, rosiglitazone was associated with higher incidence of adverse cardiovascular events such as congestive heart failure, MI, and death [92].

These trials have been very important in providing evidence to support the decisions made in choosing therapy. Studies like ADOPT which compare a TZD with commonly used antidiabetic drugs provide valuable new information to help guide future treatments. Although rosiglitazone is associated with the adverse effect of edema and weight gain, metformin and glyburide are also associated with gastrointestinal effects and weight gain respectively. However, it was shown that hyperglycemia associated with diabetes can be slowed using TZD.

Despite such positive effects, the contradictory results of trials, as well as the adverse effects of TZDs, most important being development of congestive heart failure, have limited the use of TZDs. On one hand, TZDs has a role in increasing the incidence of heart failure due to fluid retention. On the other hand, studies like PROACTIVE also suggest a beneficial role of TZDs in reducing cardiovascular disease. Similarly, DREAM trial showed an increase in heart failure but ADOPT trial showed no difference in the incidence of heart failure between rosiglitazone and other antidiabetic drugs.

## 14. New PPAR*γ* Activators and Future Therapeutic Measures

Dual PPAR alpha and *γ* activators are being developed to combine the HDL-C raising and triglyceride lowering effect of PPAR alpha with the insulin sensitivity increasing the effect of PPAR*γ*. PPAR*α* activation upregulates the genes responsible for fatty acid transport and activation. Ligands for PPAR*α* are used to treat hyperlipidemia. In this regard, Glitazar class of drugs were developed which activated both the isoforms of PPAR but the increased incidence of adverse effects prevented further research [93, 94]. Another dual activator by the name of aleglitazar has completed phase III trials [95]. The study SYNCHRONY, a phase II randomized trial to determine the cardiovascular disease risk of aleglitazar in type 2 diabetic patients [96], demonstrated a significant decrease in levels of HbA1c as well as on levels of triglyceride and LDL. In addition, an increase in HDL cholesterol was also found. However, in the phase III study known as ALECARDIO, a randomized clinical trial to determine the protective effect of aleglitazar in type 2 diabetic patients who have suffered an acute coronary syndrome did not find a reduction in the incidence of myocardial infarction or cardiovascular death with the use of aleglitazar. On the contrary, an increase in the risk of heart failure, bone fractures, gastrointestinal hemorrhage and renal function was observed.

Currently, the development of partial PPAR*γ* agonists is underway in the hopes that unlike TZDs which are full PPAR*γ* agonists, selective partial agonists will be associated with lesser adverse effects such as fluid retention, heart failure, and so forth while retaining the insulin sensitizing effects. New specific PPAR*γ* agonist S26948, displays a reduction in atherosclerotic lesions along with anti-diabetic effects [97]. INT-131 besylate is another selective peroxisome proliferator activated receptor *γ* modulator (SPPARM) evaluated in a study to determine its safety and efficacy in type 2 diabetes mellitus. A reduction in the fasting plasma glucose was observed without fluid retention or weight gain when compared to a similar model of rosiglitazone. At a dosage of 1 mg the fasting glucose reduced from 163 to 142 mg/dL without any change in the levels of NEFA, adiponectin or fasting glucose. Upon increasing the dose to 10 mg the reduction in glucose was from 183 to 137 mg/dL with significant lowering of NEFA, adiponectin, and insulin. However, an increase in weight gain and edema, as well as decrease in hematocrit levels, was also observed a phase II study using the 10 mg dosage, therefore, the advantage of using partial PPAR*γ* activators is yet to be determined with further studies [98]. SR1664 is a new anti-diabetic compound without the side effects of fluid retention and weight gain. Similar to rosiglitazone it blocked the phosphorylation of PPAR*γ* via CDK5 and is classified as a non-agonist inhibitor of CDK5 without adipogenic function in vitro [99].

The effect of a new TZD, Rivoglitazone, on the control of lipids and glucose has been compared to pioglitazone in a double-blind randomized control trial in Chinese patients with type 2 diabetes. It has been reported to be a safe and efficacious TZD-associated with improvement in glycemic control but further studies are still to be conducted [100]. Another new TZD PPAR*γ* partial agonist, balaglitazone is currently under evaluation in phase III clinical trial in US and Europe which shows glycemic control as an add-on to insulin therapy with a lower incidence of fat accumulation and fluid retention when compared to pioglitazone [101]. A new dual PPAR*α* and PPAR*γ* agonist known as Saroglitazar, with a higher activity for PPAR*α* and moderate PPAR*γ* activity, has been approved for the treatment of type 2 diabetes in India in order to control diabetic dyslipidemia [102]. However, further studies are still to be conducted before any conclusions can be made on its effect on the cardiovascular system.

## 15. Conclusions

PPAR*γ* plays an important role in cardiovascular diseases but much research is still needed to establish its function in the cardiovascular system. PPAR*γ* agonists confer benefits in diabetes and atherosclerosis, known risk factors associated with cardiovascular disease. They are beneficial as therapeutic agents resulting in improved insulin resistance, reduced glucose levels in the blood as well as reduced inflammation. However, they also have deleterious effects such as increased higher risk incidence of congestive heart failure. Therefore, their use is limited in clinical settings. At present, the use of PPAR*γ* agonists among patients is at the discretion of the physicians. Further research on PPAR physiology and pharmacology, as well the knowledge gained by the use of PPAR*γ* agonists, both known and under development, should assist in the development of newer and safer therapeutic agents.
